# Effect of Methotrexate in the Treatment of Distal Interphalangeal Joint Extensor Tendon Enthesopathy in Patients with Nail Psoriasis

**DOI:** 10.3390/jcm7120546

**Published:** 2018-12-14

**Authors:** Magdalena Krajewska-Włodarczyk, Agnieszka Owczarczyk-Saczonek, Waldemar Placek, Maja Wojtkiewicz, Joanna Wojtkiewicz

**Affiliations:** 1 Department of Rheumatology, Municipal Hospital in Olsztyn, 10-900 Olsztyn, Poland; 2 Department of Internal Medicine, School of Medicine, Collegium Medicum, University of Warmia and Mazury, 10-900 Olsztyn, Poland; 3 Department of Dermatology, Sexually Transmitted Diseases and Clinical Immunology, School of Medicine, Collegium Medicum, University of Warmia and Mazury, 10-900 Olsztyn, Poland; aganek@wp.pl (A.O.-S.); w.placek@wp.pl (W.P.); 4 Faculty of Earth Sciences, Department of Geomatics and Cartography Nicolaus Copernicus University, 87-100 Toruń, Poland; maja.wojtkiewicz@umk.pl; 5 Department of Pathophysiology, School of Medicine, Collegium Medicum, University of Warmia and Mazury, 10-900 Olsztyn, Poland; joanna.wojtkiewicz@uwm.edu.pl

**Keywords:** nail ultrasound, nail psoriasis, enthesopathy, methotrexate

## Abstract

To assess the effect of methotrexate on the development of distal interphalangeal joint extensor tendon enthesopathy in psoriasis, thirty-two people aged 34 to 57 years with nail psoriasis and distal interphalangeal joint extensor tendon enthesopathy (19 patients with Ps (psoriasis) and 13 with PsA (psoriatic arthritis) were started on methotrexate at 15 to 25 mg/week and the treatment was continued for 6 months). A total of 319 nails were examined. After six months of treatment, the thicknesses of the nail plate, nail bed and nail matrix were found to decrease in both groups of patients. Methotrexate treatment resulted in a decrease in the joint extensor tendon thickness only in patients with Ps (0.94 ± 0.05 vs. 0.96 ± 0.04, *p* < 0.001), where the tendon thickness after treatment correlated with the matrix thickness (*r* = 0.337, *p* = 0.018) and with the bed thickness (*r* = 0.299, *p* = 0.039). Methotrexate treatment resulted in a decrease in the extensor tendon thickness only in patients with Ps but not in PsA. The findings of this study may suggest the effectiveness of systemic treatment of nail psoriasis in patients without arthritis and the use of US nail examinations in Ps and PsA patients in morphological change assessment and response to treatment.

## 1. Introduction

Nail psoriasis is one of the risk factors for psoriatic arthritis (PsA) [1], with distal interphalangeal (DIP) joints being especially affected [2]. Nail changes in psoriasis have been observed in 10–55% of patients without arthritis and in up to 80% in PsA patients [3,4]. The severity and clinical manifestations of nail psoriasis depend on the anatomical structures involved in the disease process: matrix, nail bed and nail plate [3]. The anatomical conditions of the nail apparatus facilitate the spread of nail psoriasis-related inflammation to adjacent structures, including DIP joints and digital extensor tendons [4]. It seems that synovitis in the course of PsA is a secondary process to ongoing inflammation in adjacent entheses. In the development of arthritis associated with nail psoriasis and extensor tendon enthesitis, among others, locally released inflammatory mediators [4] and an abnormal pattern of vascularity observed in the synovium in PsA can participate [5].

Enthesopathies are among the most characteristic features of psoriatic arthritis [5,6]. These changes, especially located in the Achilles tendon, have been an object of interest of many studies and tend to be quite commonly assessed by imaging. However, there are few reports on the imaging of the nail apparatus. In everyday practice, assessment of psoriatic changes in nails is based mainly on clinical indices, such as nail psoriasis severity index (NAPSI) and modified NAPSI (mNAPSI) [7,8], while imaging examinations, such as ultrasonography (US) [9], magnetic resonance imaging (MRI) [10] and optical coherence tomography [11] are performed much less frequently. Owing to its relative availability, US can become a useful method in the assessment of changes in nails and adjacent structures and of the effects of treatment (Figure S1). Early diagnosis of psoriatic arthritis and the therapeutic decisions based on such diagnosis have an obvious effect on the prognosis.

According to the severity of the disease, the treatment of nail psoriasis involves different types of medications, from topical therapy to systemic therapy. There is no standardized treatment regimen for the treatment of nail psoriasis and the choice of therapy for nail psoriasis is a dilemma. Methotrexate is widely used in the treatment of various clinical manifestations of psoriasis and psoriatic arthritis; however, there are limited data about the effects of methotrexate on nail psoriasis. To date, no ultrasound evaluation of the efficacy of methotrexate treatment in inflammation of the structures of nail apparatus, including the distal interphalangeal joint extensor tendon, has been performed.

The aim of the study was to undertake ultrasonography to assess the effects of methotrexate treatment on development of enthesopathy at the DIP extensor enthesis and morphological changes in the nail apparatus in patients with psoriasis and with psoriatic arthritis with affected nails. The hypothesis tested was that the effects of methotrexate treatment of DIP extensor enthesitis might differ between psoriatic patients and those with psoriatic arthritis. Additionally, we hypothesized that methotrexate may affect the ongoing inflammation in the anatomical structures of the nail apparatus.

## 2. Materials and Methods

This study was a prospective observational study in which patients with nail psoriasis and distal interphalangeal enthesitis were started on methotrexate. 

The study lasted from January to September 2018 and was carried out at the Department of Rheumatology and the Department of Dermatology of the University of Warmia and Mazury in Olsztyn (Poland). 

Thirty-two consecutive patients with psoriatic changes in nails in whom a US examination revealed DIP joint extensor tendon enthesopathy in at least one finger were recruited (19 patients with Ps and 13 with PsA). A total of 319 nails were examined. During the study, all patients were started on methotrexate administered orally and the treatment was continued for 6 months. The decision to start the treatment was based on the clinical picture of the skin or joint disease only. Methotrexate doses given to the patients ranged from 15 to 25 mg/week. Patients were assessed at baseline and 6 months.

Inclusion criteria: patients with nail psoriasis in at least one finger and extensor tendon enthesitis observed in a US examination in at least one distal interphalangeal joint.

Exclusion criteria: psoriatic patients diagnosed with osteoarthritis of the hands, those who work hard physically and those with changes in nails other than caused by psoriasis were excluded from the study. Patients previously treated with methotrexate were excluded from the study as those receiving steroids or other disease-modifying drugs (DMARDs) at the time of recruitment for the study.

The patients’ age ranged from 34 to 57 years.

Psoriatic arthritis was diagnosed based on CASPAR criteria [12].

All of the patients were examined by an experienced dermatologist. The macroscopic progression of psoriatic changes in nails with pitting, with hyperkeratosis and/or onycholysis was assessed by mNAPSI. The intensity of psoriatic changes in the skin was assessed with the PASI (Psoriasis Area and Severity Index) [13].

The activity of psoriatic arthritis was assessed by the Disease Activity Score calculated for 28 joints (DAS 28) [14] and the number of tender (tender joint count—TJC) and swollen (swollen joint count—SJC) joints, calculated for 68 and 66 joints, respectively. 

A US examination of nails and distal interphalangeal (DIP) joint extensor tendons was conducted in all of the patients twice at an interval of six months. The examination was conducted by a rheumatologist experienced in ultrasound examinations of the skeletal and muscular system. Morphological changes were examined with DermaMed equipment and software (Dramiński, Olsztyn, Poland) with a linear head and a variable frequency ranging from 12 to 48 MHz. All nail examinations (558 nails, 2 nails were excluded because of a previous injury) were conducted at 24 MHz. An assessment of intensified blood supply, corresponding to intensification of inflammation, was made with a Mindray M5 (Mindray, Guangdong, China) apparatus with the Power Doppler (PD) technique. An assessment of the nails, extensor tendons and DIP joints was made by placing the head on the dorsal side. To avoid pressure on surface tissues, an appropriate amount of gel without gel pads was used. The intensified blood flow, made visible by the PD technique, was confirmed by pulsed wave Doppler spectrum. The nail thickness was measured as the maximum distance between the dorsal and ventral nail plates. The nail bed thickness was measured as the distance between the ventral plate and the bone margin of the distal phalanx. The nail matrix thickness was measured at the proximal end of the nail bed. 

According to the classification proposed by Wortsman et al. [15], morphological changes in nails in US examinations were described as: focal hyperechoic involvement of the ventral plate (type I), loosening of the borders of the ventral plate (type II), wavy plates (type III) and loss of definition of both plates (type IV).

Entheses were assessed in accordance with OMERACT (Outcome Measures in Rheumatology) [16] recommendations in a US examination, on the scale of greyness, at the place where the extensor tendon is attached to the distal phalanx of the DIP joint. Loss of normal fibrillary architecture, thickened tendon or enthesophytes at its bony insertion and bony changes, including erosions, were regarded as enthesopathies. The tendon thickness was measured at the place where it is attached to the distal phalanx. Inflammation and inflammation-associated locally increased vascularization was assessed with the PD and verified by a pulsed-wave Doppler spectrum. 

Inflammatory markers were measured with two standard laboratory parameters: erythrocyte sedimentation rate assessed using BD Vacutainer Sedi-15 equipment (BD, Franklin Lakes, NJ, USA) and the concentration of C-reactive protein measured with a standard immunoturbidimetric method using a COBAS 6000 INTEGRA apparatus (Roche Diagnostics, Mannheim, Germany).

The study was conducted according to the Good Clinical Practice guidelines. Each participant provided written consent to participation in the study and was coded with a unique ID. The results of analyses were saved by indexing with ID code only, personal data with individual IDs were saved in an additional file. The study was approved by the Bioethics Committee of the Warmia and Mazury Chamber of Physicians (OIL 625/16/Bioet; 21.12.2016). The study was registered on ClinicalTrials.gov (protocol ID: UWM/Ps-ENTH.2018/001; ClinicalTrials.gov ID: NCT03757364).

## 3. Statistical Analysis

StatSoft program, Inc. STATISTICA, version 12.5 (StatSoft, Tulsa, OK, USA) was used for calculations. The obtained results were presented as an arithmetic mean and standard deviation. The Mann-Whitney *U*-test and the Kruskal-Wallis test were used for comparative analysis between the groups. The presence of the relationship between quantitative features was tested using Pearson’s correlation coefficient for parameters consistent with normal distribution and Spearman’s correlation coefficient in case of non-compliance with normal distribution. Linear regression modelling was used to evaluate the relationships between the studied data. Variable models were selected stepwise using backward elimination. The statistical level of significance was *p* < 0.05.

## 4. Results

Altogether, 32 methotrexate-naive patients with nail psoriasis, aged 34–57 years, participated in the study (19 with psoriasis without arthritis and 13 with psoriatic arthritis). There was no difference between the groups regarding age or sex. Significantly higher inflammation parameters were observed in patients with arthritis. No differences were observed in both groups regarding the intensity of changes in nails as assessed with the mNAPSI or skin changes as assessed with the PASI. The duration of psoriasis in both the groups of patients did not differ significantly (Table 1).

**Figure 1:**
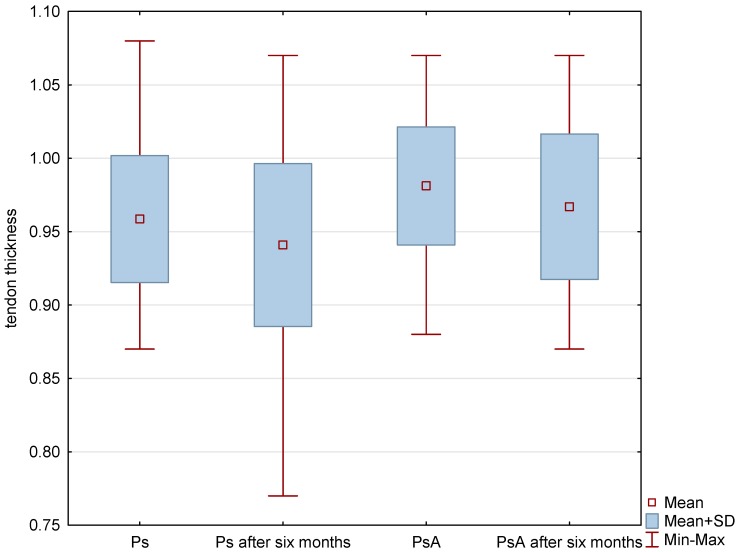
US measurements of tendon thickness in patients studied before and after methotrexate therapy. *p* < 0.001 Ps before therapy vs. PsA before therapy. *p* < 0.001 Ps before treatment vs. Ps after six-month therapy. *p* = 0.61 PsA before therapy vs. PsA after six-month therapy. Ps: psoriasis, PsA: psoriatic arthritis.

**Figure 2:**
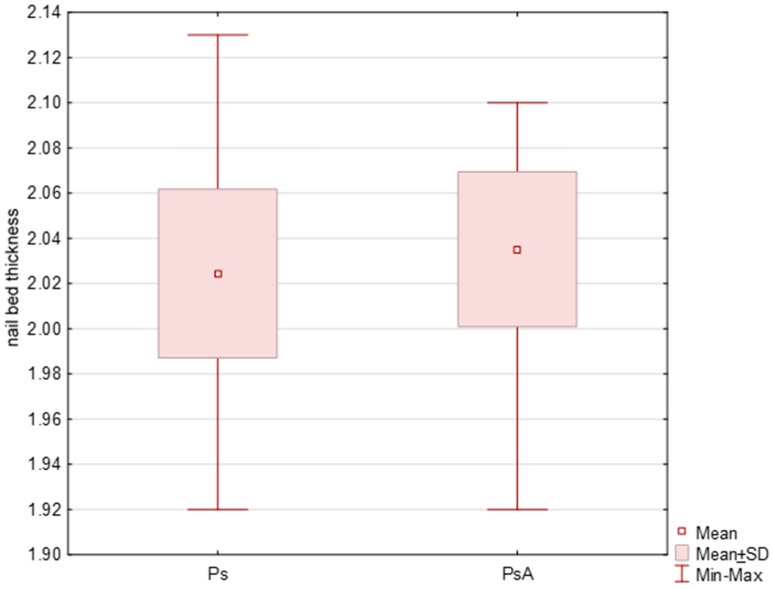
Initial US measurements of nail bed thickness in patients studied. *p* < 0.001. Ps: psoriasis, PsA: psoriatic arthritis.

A total of 319 nails were examined: 190 nails in patients with psoriasis without arthritis and 129 nails in patients with PsA. Psoriatic changes in patients with Ps and PsA were present in 145 (76%) and 96 (74%) nails, respectively. Both the extensor tendon (Figure 1) and nail bed (Figure 2) in patients with PsA were thicker than in patients with psoriasis without arthritis (in both cases *p* < 0.001) and no statistical differences were observed in the thickness of nail plates and nail matrix. The nails with psoriatic changes were assessed in a US examination in regard to their morphology, in accordance with the classification proposed by Wortsman et al. (Figure S2). Focal hyperechoic involvement of the ventral plate (type I), loosening of the borders of the ventral plate (type II) and wavy plates (type III) in patients with psoriasis was observed in 83%, 11.5% and 5.5% of the nails under examination, respectively. No loss of definition of both plates (type IV) was observed. Type I, II, III and IV changes were present in 15.5%, 68%, 7% and 1.5% of the patients with PsA, respectively (Table 2). The digital extensor tendon was the thickest in type II of the changes in US in patients with Ps (*p* = 0.019), whereas no difference in the thickness of the extensor tendon was found in the group with arthritis depending on the type of changes of nail plates in an US examination. The thickness of the digital extensor tendon did not differ in patients with Ps and with PsA with the onycholysis and hyperkeratosis type changes (concomitant or existing separately), but it was significantly greater than in the pitting-type of changes (*p* = 0.041 and *p* = 0.033, respectively). A correlation was found in patients with Ps and PsA between the intensity of clinical changes in nails, as assessed with mNAPSI, with the thickness of the extensor tendon (*r* = 0.299, *p* = 0.042 vs. *r* = 0.336, *p* = 0.019, respectively). The tendon thickness in patients with Ps correlated with the matrix thickness (*r* = 0.346, *p* = 0.023) and the nail bed thickness (*r* = 0.285, *p* = 0.034), whereas in patients with PsA, the tendon thickness correlated with the nail bed thickness (*r* = 0.401, *p* = 0.011). No relationship between the thickness of the digital extensor tendon in a DIP joint with the intensity of skin changes was observed in either of the groups.

Because of the aim of the study, enthesopathies of DIP joint tendons in at least one digit were observed in a US examination. In patients with Ps and PsA enthesopathies were present more frequently in digits with nail involvement than in those in which nails were not affected. Increased vascularization as assessed by the PD technique within entheses under study in both groups was observed more frequently in digits with psoriatic nails than in those with no clinical changes in the nails. More frequent presence of enthesopathies and increased PD signal were observed in PsA patients. (Table 3)

The tendon thickness in Ps and PsA increased with the duration of psoriasis (*r* = 0.318, *p* = 0.037 vs. *r* = 0.301, *p* = 0.024, respectively), and in patients with PsA and with the duration of arthritis (*r* = 0.409, *p* = 0.019). In the group with arthritis, an increase in the thickness of the digital extensor tendon was correlated with the swollen joint count (*r* = 0.321, *p* = 0.025), but it was not correlated with the tender joint count or the disease activity as measured by the DAS 28. 

**Figure 3:**
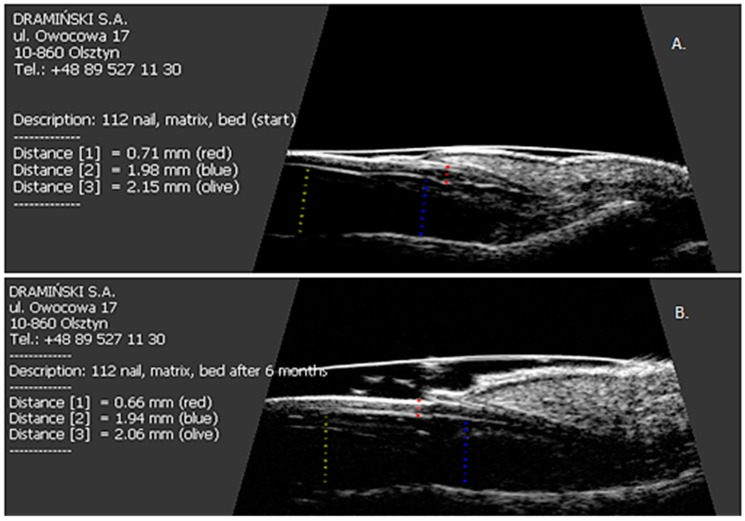
The effect of six months of methotrexate treatment on the thickness of the nail plate, nail bed and nail matrix in patients studied. (**A**) Before treatment; (**B**) after six months of therapy.

**Figure 4:**
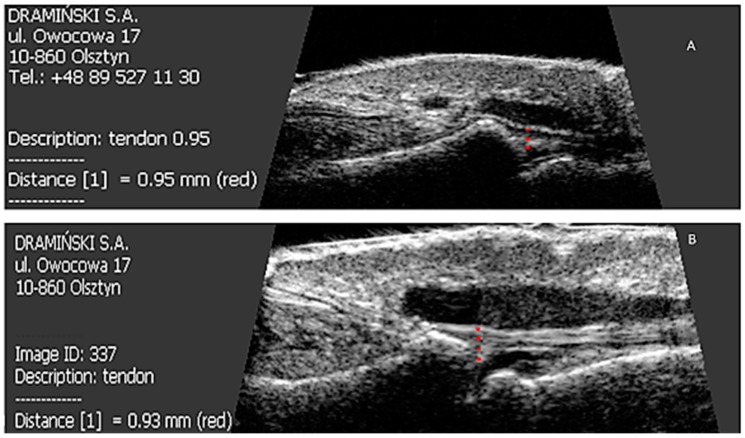
Effect of six months of methotrexate treatment on the thickness of the finger extensor tendon in the distal interphalangeal joint in patients studied. (**A**) Before treatment; (**B**) after six months of therapy.

All patients were started on methotrexate after the first US examination. After six months of treatment, a decrease in the thickness of nail plates, nail beds and nail matrix was observed in both study groups (Table 4 and Table 5, Figure 3). Methotrexate treatment in the group of patients without arthritis reduced the extensor tendon thickness (Table 4, Figure 1), whereas no such effect was observed in patients with PsA (Table 5, Figure 1 and Figure 4). Methotrexate treatment in both groups reduced the intensity of vascularization as assessed with PD at entheses under study. An intensified PD signal in Ps patients after treatment was observed in 42/190 (22%) nails, like in the PsA group, where it was 29/129 (22.5%) nails.

A regression of nail changes, as assessed with mNAPSI, was observed in all the Ps and PsA patients (Figure S3). Decreased intensity of clinical changes after methotrexate treatment was correlated with decreasing extensor tendon thickness (*r* = 0.306, *p* = 0.037) in patients with Ps. Rather unexpectedly, no such relationship was observed in patients with PsA. In patients with Ps treated with methotrexate, the tendon thickness after six months still correlated with the matrix thickness (*r* = 0.337, *p* = 0.018) and the nail bed thickness (*r* = 0.299, *p* = 0.039). The tendon thickness in the PsA patients who received the treatment did not correlate with the thickness of the other nail structures.

When the linear regression model was applied, which consisted of taking into account in the original model of all potential variables (selected stepwise with backward elimination), the factors were determined which, in combination, had the greatest effect on the thickness of the digital extensor tendon in the DIP joint in the patients after treatment with methotrexate. After the therapy, the tendon thickness in psoriatic patients with no arthritis was found to be affected by the initial thickness of the nail bed and the nail matrix, duration of the skin disease and initial CRP (C-reactive protein) concentration (Table 6). The tendon thickness in the PsA patients was found to be significantly affected by the duration of arthritis, the tender joints’ count and the swollen joints’ count at the beginning of the study (Table 7).

## 5. Discussion

Psoriatic changes in nails are a known risk factor for the development of arthritis, and enthesopathies are among the most common non-articular symptoms of spondyloarthropathies, including psoriatic arthritis [3,17]. 

In the current study, a US assessment was made of patients with existing DIP joint extensor enthesopathies. To date, few reports have been published on a US assessment of the nail apparatus [18,19,20,21]. We have previously used ultrasound to show a high frequency of subclinical entheseal involvement in DIP joint in patients with Ps [21]. In our previous study in patients with Ps and PsA, loss of normal fibrillary architecture, enthesophytes, and bony changes were observed in 31% and 68% digits with nail involvement, respectively [21]. In a study by Castellanos-González et al., the frequency of changes in the digital extensor tendon or its attachment in the DIP joint of digits with nail involvement was nearly 83% [22]. In a paper by Acosta-Felquer et al., the frequency of enthesopathies in patients with psoriasis and PsA in a US examination of digits with nail involvement did not differ and was 61% and 60%, respectively [19]. More frequent presence of enthesopathies in other sites in patients with psoriasis with no clinical symptoms of arthritis has been described in the literature [23,24]. Ash et al. described the relationship between the occurrence of psoriatic changes in nails and the intensity of enthesopathies in joints other than DIP [18]. 

In both groups of patients in our study, as in a study by Castellanos-González et al. [22], the thickness of the DIP joint extensor tendon increased more when onycholysis and/or hyperkeratosis was present than when pitting-type changes occurred. In a study by Aydin et al. [25], the tendon thickness additionally depended on the existence of the pitting symptom. In the current study, no correlation between the nail plate thickness and the tendon thickness was found. Apart from nail plates, we conducted an ultrasound assessment of the nail bed and matrix. The digital extensor tendon thickness correlated with the bed thickness in both groups under study and it also correlated with the matrix thickness in the group of patients with Ps. Aydin et al. did not find such a correlation, but the thickness of the digital extensor tendon as measured in their study correlated with the skin thickness as assessed above the DIP joint [25]. 

Methotrexate is the most frequently used conventional disease-modifying antirheumatic drug for the treatment of PsA, but data on the use of methotrexate in PsA are equivocal. Both reduction in disease activity and no improvement in patients with PsA on methotrexate monotherapy were observed [26]. The treatment of enthesitis is also a challenge since conventional DMARDs, including methotrexate may be not significantly effective [27]. There is no standardized treatment regimen for the treatment of nail psoriasis. The use of methotrexate in psoriatic nails is less described than its use in skin psoriasis. Both oral and injection therapies are possible. Cases of effective treatment of nail psoriasis with intralesional methotrexate have been described [28,29]. In a recently published study, topical administration of methotrexate proved to be more effective in the treatment of nail psoriasis than triamcinolone or ciclosporin, with fewer side effects [30]. Moderate effectiveness of systemic therapy with methotrexate on nail psoriasis was described by Gümüşel et al. [31]. In a case-report by Lee, oral methotrexate was successfully used at low doses (5 mg/week) in severe psoriatic nail dystrophy involving all 20 nails [32]. There is no data available on the effectiveness of methotrexate in the treatment of distal interphalangeal joint extensor tendon enthesopathy in patients with nail psoriasis. In a study by Litinsky et al., no change on US was observed in the thickness of the extensor and flexor tendons of the second and third fingers of both hands measured above the MCP joint line before and after 3 months of therapy with methotrexate in PsA patients [33]. In our study, methotrexate treatment resulted in a decrease of the extensor tendon thickness only in patients with Ps but not in PsA. This may be attributed to the more frequent occurrence of chronic changes in patients with PsA, which include—apart from thicker tendon resulting from active inflammation—transformation of the tendon with the loss of the fibrillary architecture and the presence of osteophytes and other bone transformations at the sites of attachment. Unlike inflammatory changes in Ps, such fixed changes may not have been susceptible to methotrexate. In our study, using a linear regression model, we identified several factors that could be potential prognostic factors of weak response to methotrexate treatment. In psoriatic patients, the tendon thickness after the therapy was found to be influenced by the initial thickness of the nail bed and the nail matrix, reflecting local inflammation, initial CRP concentration, reflecting systemic inflammation, and duration of the skin disease. In the PsA group the tendon thickness was found to be significantly affected by the duration of arthritis, the tender joints’ count and the swollen joints’ count at the beginning of the study. 

Increased vascularization may be a symptom of ongoing open or subclinical inflammation. In the current study, increased vascularization around the digital extensor tendon in the DIP joint, as assessed by the PD technique, was observed in the Ps and PsA patients following methotrexate treatment less frequently than before the treatment started (22% vs. 62% and 22.5% vs. 70%, respectively). An intensified PD signal was observed in the extensor tendon area more frequently in DIP joints of fingers with existing changes in the nails in both groups of patients, similar to Acosta-Felquer et al. [19]. In a study by Sandobal et al., increased vascularization in DIP joints was observed much more frequently in patients with PsA than in those with Ps, irrespective of the existing changes in nails [20], unlike in the study by Acquiter et al., where no difference regarding the PD signal in DIP joints was observed in Ps and PsA patients between those with and without nail involvement [34]. 

In our study, no US improvement after therapy was observed in deviations involving bone changes (erosions and enthesophytes) or calcifications.

All of this can justify the early use of methotrexate in patients with nails affected by psoriasis as a drug potentially inhibiting inflammation within the extensor tendon and development of DIP joint arthritis, but not affecting chronic enthesopathies. Such a therapeutic approach certainly requires further study.

Potential limitations of this study include (among others) the fact that existing changes in nails could not be hidden during US examinations, which made full blinding of the study impossible.

## 6. Conclusions

The effects of methotrexate treatment of DIP extensor enthesitis differ between Ps patients and PsA patients. Methotrexate treatment resulted in a decrease in the extensor tendon thickness only in patients with Ps but not in PsA. The potential prognostic factors of weak response to methotrexate treatment in psoriatic patients increased initial thickness of the nail bed and the nail matrix, increased initial CRP concentration and duration of the skin disease. In the PsA group weak response to the therapy was significantly influenced by the duration of arthritis, the tender joint count and the swollen joint count at the beginning of the study. The findings of this study may suggest the reasonableness of early systemic treatment of nail psoriasis with methotrexate as the psoriatic enthesopathy prevention, as well as the use of US nail examinations in Ps and PsA patients in the assessment of morphological changes and the response to treatment. Obviously, the use of such US examinations must be confirmed in further studies.

## Figures and Tables

**Table 1:** Age and clinical characteristics of the patients.

	Ps (*n* = 19)	PsA (*n* = 13)	*p*
male/female (number)	8/11	6/7	
Age (years)	45.6 ± 9.6	46.1 ± 8.8	0.307
Ps duration (years)	15.1 ± 10.3	16.3 ± 6.6	0.631
PsA duration (years)	-	6.9 ± 7.5	-
DAS 28	-	3.3 ± 0.5	-
PASI	6.1 ± 3.6	5.6 ± 3.8	0.087
mNAPSI	21.1 ± 15.7	20.5 ± 16.1	0.154
TJC	-	2.6 ± 1.2	-
SJC	-	2.0 ± 0.4	-
CRP (mg/dL)	2.7 ± 1.6	9.1 ± 3.5	*p* < 0.001
ESR	11.6 ± 4.6	23.5 ± 8.3	*p* < 0.001

Results are presented as mean values and standard deviations (SD). Ps: psoriasis, PsA: psoriatic arthritis, DAS: disease activity score, PASI: psoriasis area severity index, mNAPSI: modified nail psoriasis severity index; ESR: erythrocyte sedimentation rate, CRP: C-reactive protein, TJC: tender joint count; SJC: swollen joint count.

**Table 2:** Wortsman classification of the psoriatic nails studied.

Wortsman Classification	Ps (*n* = 174)	PsA (*n* = 100)
I	144	15
II	10	74
III	7	9
IV	-	2

Results are presented as numbers. Ps: psoriasis, PsA: psoriatic arthritis.

**Table 3:** Total number of ultrasound findings.

	Ps *n*/*n* of Fingers Studied (%)	PsA *n*/*n* of Fingers Studied (%)	*p*
Enthesopathies	124/190 (65%)	94/129 (72%)	0.014
Enthesopathies in fingers with Ps nails	118/174 (68%)	89/100 (89%)	<0.001
Enthesopathies in fingers with no Ps changes	6/16 (37%)	16/29 (55%)	0.006
Increased PD signal	118/190 (62%)	90/129 (70%)	0.021
Increased PD signal in fingers with Ps nails	110/174 (63%)	83/100 (83%)	<0.001
Increased PD signal in fingers with no Ps changes	7/16 (43%)	17/29 (28%)	0.018

Results are presented as numbers and %. Ps: psoriasis, PsA: psoriatic arthritis, PD: Power Doppler.

**Table 4:** US measurements of the fingers in patients with psoriasis.

	Ps (190/319) Initial	Ps (190/319) after 6 Months of Mtx Treatment	*p*
NP thickness (mm)	0.74 ± 0.04	0.73 ± 0.04	0.004
NB thickness (mm)	2.02 ± 0.03	2.0 ± 0.05	<0.001
Matrix thickness (mm)	1.93 ± 0.02	1.93 ± 0.03	<0.001
Tendon thickness (mm)	0.96 ± 0.04	0.94 ± 0.05	<0.001

Results are presented as mean values and standard deviations (SD). NP: nail plate; NB: nail bed; Ps: psoriasis: Mtx: methotrexate.

**Table 5:** US measurements of the fingers in patients with psoriatic arthritis.

	PsA (129/319) Initial	PsA (129/319) after 6 Months of Mtx Treatment	*p*
NP thickness (mm)	0.75 ± 0.05	0.74 ± 0.05	0.002
NB thickness (mm)	2.04 ± 0.03	2.01 ± 0.06	<0.001
Matrix thickness (mm)	1.93 ± 0.01	1.93 ± 0.01	0.002
Tendon thickness (mm)	0.98 ± 0.04	0.97 ± 0.05	0.061

Results are presented as mean values and standard deviations (SD). NP: nail plate; NB: nail bed; PsA: psoriatic arthritis, Mtx: methotrexate.

**Table 6:** The regression coefficients in modelling for finger extensor tendon thickness in psoriatic patients treated with methotrexate.

	NB Thickness (At the Beginning of the Study)	Matrix Thickness (At the Beginning of the Study)	Ps Duration	CRP (At the Beginning of the Study)	Adjusted *R*^2^
Ps (fingers, *n* = 190)	0.4722 (0.0000)	0.4410 (0.001)	0.3921 (0.015)	0.3641 (0.017)	0.386

The *p*-value is shown in brackets. Ps: psoriasis; NB: nail bed; CRP: C-reactive protein.

**Table 7:** The regression coefficients in modelling for finger extensor tendon thickness in psoriatic arthritis patients treated with methotrexate.

	PsA Duration	TJC (At the Beginning of the Study)	SJC (At the Beginning of the Study)	Adjusted *R*^2^
PsA (fingers, *n* = 129)	0.4648 (0.0000)	0.4111 (0.0000)	0.4003 (0.0000)	0.317

The *p*-value is shown in brackets. PsA: psoriatic arthritis, TJC: tender joints count; SJC: swollen joints count.

## Supplementary Materials

The following are available online at http://www.mdpi.com/2077-0383/7/12/546/s1, Figure S1: Nail of a healthy person, Figure S2: Longitudinal scan of psoriatic nail, Figure S3: The effect of six months of methotrexate treatment on the clinical improvement of nail psoriasis.
