# Supplementary material for: Effect of Methotrexate in the Treatment of Distal Interphalangeal Joint Extensor Tendon Enthesopathy in Patients with Nail Psoriasis
Source: J Clin Med. 2018 Dec 14;7(12):546. doi: 10.3390/jcm7120546 (PMC6306839; doi:10.3390/jcm7120546)
Supplement: Supplementary file 1 [file jcm-07-00546-s001.pdf]

Journal of
Clinical Medicine

MDPI

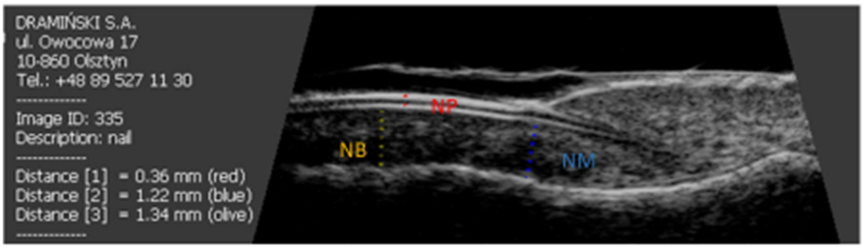

Figure S1. Nail of a healthy person. NP: nail plate; NB: nail bed; NM: nail matrix.

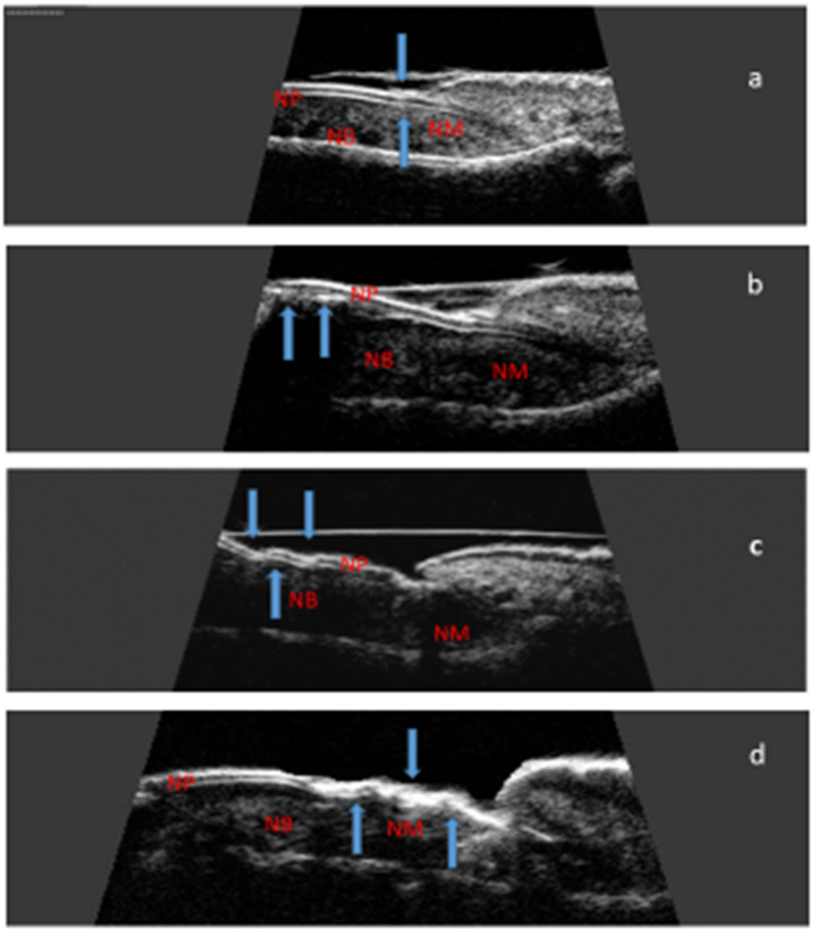

Figure S2. Longitudinal scan of psoriatic nail. (**a**) Focal hyperechoic involvement of the ventral plate, (**b**) Loosening of the borders of the ventral plate, (**c**) Wavy plates. d Loss of definition of both plates. NP: nail plate; NB: nail bed; NM: nail matrix.

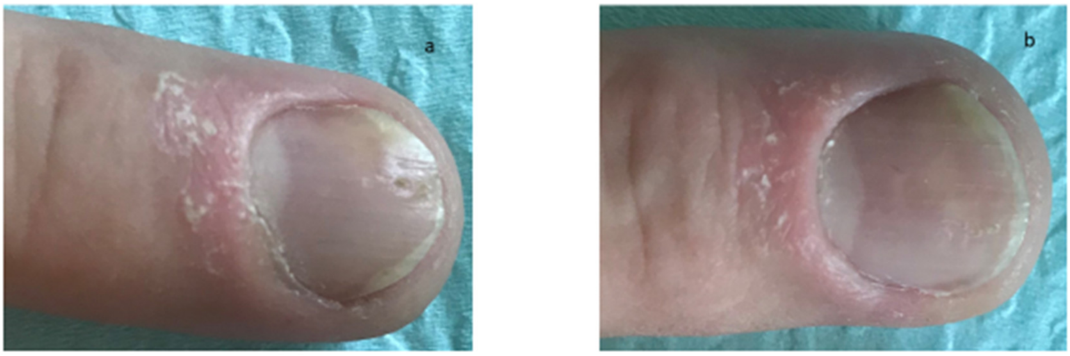

Figure S3. The effect of six months of methotrexate treatment on the clinical improvement of nail psoriasis. (**a**) before treatment, (**b**) after 6 months of treatment with methotrexate.
